# Management of Diabetes Mellitus in Patients with Chronic Liver Diseases

**DOI:** 10.1155/2019/6430486

**Published:** 2019-12-14

**Authors:** Yingying Zhao, Huichun Xing, Xiaomei Wang, Weini Ou, Hong Zhao, Ben Li, Yue Li, Ying Duan, Liwei Zhuang, Wei Li, Danying Cheng, Min Quan, Yu Zhang, Shibo Ji

**Affiliations:** Department of Hepatology, Division 3, Beijing Ditan Hospital, Capital Medical University and Teaching Hospital of Peking University, 8 Jingshundong Street, Beijing 100015, China

## Abstract

Diabetes mellitus (DM) is a common chronic disease affecting humans globally. During the last few years, the incidence of diabetes has increased and has received more attention. In addition to growing DM populations, DM complications are involving injuries to more organs, such as the heart and cerebral vessel damage. DM complications can reduce quality of life and shorten life spans and eventually also impede social and economic development. Therefore, effective measures to curb the occurrence and development of diabetes assist in improving patients' quality of life, delay the progression of DM in the population, and ease a social burden. The liver is regarded as an important link in the management and control of DM, including the alleviation of glucose metabolism and lipid metabolism and others via glucose storage and endogenous glucose generation from glycogen stored in the liver. Liver cirrhosis is a very common chronic disease, which often lowers the quality of life and decreases life expectancy. According to a growing body of research, diabetes shows a close correlation with hepatitis, liver cirrhosis, and liver cancer. Moreover, coexistence of liver complications would accelerate the deterioration of patients with diabetes. Liver cirrhosis and diabetes influence each other. Thus, in addition to pharmacological treatments and lifestyle interventions, effective control of cirrhosis might assist in a better management of diabetes. When it comes to different etiologies of liver cirrhosis, different therapeutic methods, such as antiviral treatment, may be more effective. Effective control of cirrhosis might be a strategy for better management of diabetes.

## 1. Introduction

In 2015, there were approximately 415 million diabetes mellitus (DM) patients aged 20 to 79 years old, and researchers estimated approximately 642 million new patients within the next 2 decades [1]. Among the multiple etiologies of DM, insulin deficiency and/or insulin resistance (IR) are the most important ones. The long-term metabolic disorders include macrovascular and microvascular complications, which subsequently induce damage to multiple systems, such as cases of circulatory system dysfunction and renal impairment. Cardiovascular complications occur in most diabetic populations, whereas kidney complications more commonly occur in Asian DM patients [2]. In addition to common chronic complications of DM, severe acute complications, like diabetic ketoacidosis (DKA) or the diabetic hyperglycemic hyperosmolar state, seriously affect the clinical outcomes and at times even become life-threatening conditions [3]. In 2017, the mortality of DM was 1.37 million [3].

The liver contributes to the metabolic processes of the body and has a vital function, especially in glucose homeostasis. Glucagon in the liver promotes glycogen breakdown through a series of reactions. Glycogen synthesis is mainly regulated by insulin [4]. Recently, more and more studies have reported the association of diabetes with chronic liver diseases, cirrhosis, liver cancer, and even colorectal, pancreatic, and kidney cancers [5, 6]. Cirrhosis is the 11th most common cause of mortality, leading to 1.16 million civilian deaths per year around the world [7]. The common etiological factors of cirrhosis comprise accumulation of nonalcoholic fatty liver disease (NAFLD), viruses, and excessive alcohol consumption. There is a strong correlation between cirrhosis and hyperglycemia [8].

Unlike traditional strategies for the management of diabetes, such as moderate intensity physical activity, and lifestyle management like reducing tobacco, and pharmacological interventions [2], to delaying the progression of liver disease, seems to be vital for preventing the adverse consequences caused by DM [5]. This review explores the possible mechanisms between DM and liver diseases, as well as the distinct management of diabetes in DM patients with liver diseases.

## 2. The Interrelation between DM and Nonalcoholic or Alcoholic Fatty Liver Disease and Potential Mechanisms among Them

### 2.1. Epidemiological Characteristics of NAFLD or Alcoholic Liver Disease (ALD)

NAFLD may become one of the common reasons for liver transplantations by 2030 in Western countries [9]. Like viral liver diseases, there are several different NAFLD patterns based on their pathological features, including steatosis, nonalcoholic steatohepatitis (NASH), and cirrhosis accompanied by even hepatocellular carcinoma (HCC). According to the pathological characteristics, ALD can be divided into three stages: simple steatosis, steatohepatitis, and hepatic fibrosis or cirrhosis. The majority of ALD patients have steatosis, while only a small percentage of these patients progress to liver fibrosis [10]. The incidence of NAFLD increases year by year, likely reaching to 101 million by 2030 [7]. NAFLD influences not only the burden of liver-related morbidity and mortality, such as severely impairing hepatic structure and function, with cirrhotic liver failure and HCC, but also extrahepatic complications like DM [11].

### 2.2. Interaction between DM and NAFLD or ALD

#### 2.2.1. Effect of DM on NAFLD/or ALD

DM patients are more likely to develop advanced fibrosis and NAFLD [11]. For NAFLD patients with DM, besides an increased risk of fibrotic or cirrhotic development, the probability of suffering from hepatic malignant tumors, hospitalization, and death due to liver disease also shows an upward trend [11, 12]. According to a retrospective study conducted by McPherson et al. in 2016 in 108 NAFLD patients, DM was an independent predictor of fibrosis progression and NAFLD patients with DM also showed a more severe degree of fibrosis or cirrhosis (*P* < 0.001) [13].

A retrospective study was conducted in the United States enrolling 480 patients with fatty liver, regardless of alcohol-induced liver damage (2004–2011). The results showed that diabetic patients had a 3 times higher probability of developing cirrhosis or hepatic malignant tumors, whereas there was no obvious discrepancy in the survival probability. For diabetic NAFLD patients, the number of patients with cirrhosis or morbidity associated with cirrhosis at the beginning of the study (51 of 160 vs. 24 of 155, *P* = 0.009) was more than that for nondiabetics ones. Among ALD or NAFLD patients with diabetes, the pathogenesis of cirrhosis, hepatic encephalopathy, and liver cancer was more rapid than that among nondiabetic ones, while the other two groups showed no statistical differences in the survival rates at the end of the follow-up period [12].

#### 2.2.2. Effect of NAFLD/ALD on DM

In recent years, several studies have suggested that NAFLD typically triggers a series of extrahepatic complications, such as DM, incremental risks of colon tumor [6], CVD, chronic kidney disease (CKD), and endocrine system diseases [9, 14].

A meta-analysis containing 19 studies suggested that NAFLD increased the risk of DM (random-effects hazard ratio (HR) 2.22) in contrast to those with non-NAFLD, and the 95% confidence interval (CI) for it was 1.84-2.60; particularly, patients with cirrhosis were more prone to the progression of DM (random-effects HR 4.74, 95% CI 3.54-5.94) [15]. Another study containing 70,303 NAFLD patients without diabetes or obesity with duration of ≥7.9 years follow-up suggested that NAFLD was an independent predictor for DM even within the normal weight range [16]. With continued follow-up, 852 subjects developed DM (median interquartile range (IQR) 3.71 (2.03) years) [16]. The proportion of advanced fibrotic and cirrhotic NAFLD patients that developed T2DM was larger than that of those without fibrosis or cirrhosis (89% vs. 47%, *P* < 0.001) [13]. The effects of NAFLD-related cirrhosis on diabetes increased the risk of DM and also promoted the deterioration of diabetic complications [6, 14]. Increasingly, studies indicate that NAFLD might be an independent risk factor that has impact on CVD [9].

NAFLD-induced complications of DM also appear in type 1 diabetes mellitus (T1DM) [17, 18]. A retrospective study of 286 T1DM subjects showed that the NAFLD population had an increased risk of CVD than those without NAFLD (17.3% vs. 1.5%, *P* < 0.001). NAFLD increased the risk of CVD events (HR 8.16, 95% CI 1.9-35.1, *P* < 0.005) [17]. In addition, the incidence of CKD in T1DM patients was higher than that in those without NAFLD (HR 2.85, 95% CI 1.59-5.1; *P* < 0.001). NAFLD was considered to be a risk factor for the occurrence of CKD among T1DM patients [18].

A UK study cohort of 134,368 diabetic people with multiple fatty liver disease caused by ALD (*n* = 1707) or NAFLD (*n* = 1452) was observed at different time periods for CVD events (4.3 years) and mortality (4.7 years). This result indicated that DM patients with ALD or NAFLD who had been hospitalized were more likely to develop CVD events, cancers, and all-cause mortality than those without liver disease [6]. This correlation suggests a relation between incidence of CVD, ALD, and NAFLD. Reversal of ALD and NAFLD at the earlier stage can be achieved by lifestyle changes, such as abstinence, losing weight, and exercise [6, 8, 19].

### 2.3. The Probable Mechanisms for NAFLD/ALD

ALD is mainly caused by excessive drinking. ALD patients are majorly characterized by steatosis, but only a small percentage of these patients progresses to liver fibrosis. Genetic polymorphisms of cytochrome p450 (CYP) 2E1 [10] and other enzymes involved in ethanol metabolism might affect the severity of alcoholic steatohepatitis. Adiponectin, a protein that is associated with insulin sensitivity, is an endogenous producer of adipocytes, while hypoadiponectinemia is related to insulin resistance [10].

Approximately a quarter of ALD-related cirrhosis patients is associated with excessive drinking. The metabolites and byproducts of ethanol are metabolized directly in the pancreas, which may damage acinar cells [20, 21]. Through the activation of the inflammatory responses and endothelial dysfunction, extracellular matrix (ECM), acetaldehyde, and reactive oxygen species (ROS) are further produced and deposited, further activating the transcription factors that comprise nuclear factor *κ*B (NF-*κ*B), as well as activator protein-1 (AP-1) [21, 22].

All these changes might cause vascular cell damage and angiogenesis. In addition to these changes, necrosis or apoptosis of acinar cells, proliferative and profibrinogenic of growth factors including platelet-derived growth factor (PDGF), TGF-*α* [21] and connective growth factor (CTGF) [23] are all involved in fibrogenesis. This pathway induces chronic pancreatitis characterized by atrophy and fibrosis of acinar cells, leading to exocrine and endocrine glandular dysfunction [20, 21].

Immoderate fat deposition on the liver of patients with NAFLD attenuates insulin signaling, leading to IR and abnormal hepatic metabolism. This mechanism almost doubled the incidence of DM [9]. Besides IR, chronic diabetic vascular complications include atherosclerosis, worse blood glucose control, and imbalance of anticoagulant and procoagulant inflammatory processes, sequentially increase the occurrence of CVD events, especially among NAFLD patients with fibrosis or cirrhosis [11]. Moreover, HCC increases by means of inflammatory reactions [9]. Oxidative stress (OS) induced by ROS and inflammation play an important function, leading to liver cell death and damage of tissue [19, 22].

Increased free fatty acids (FFAs) in circulation might be vital for IR, inflammation, obesity, T2DM, and hypertension (HTN) [22, 24]. FFA-mediated inactivation of phosphoinositide 3-kinase (PI3K) [22] is associated with IR, impairing hepatic insulin signaling, in fatty tissue and the musculoskeletal system [24, 25]. In addition to the alternation of insulin signaling and FFAs, some particular proteins originating from the liver, which are also called hepatokines, are linked to the induction of metabolic dysfunction [26]. Among them, retinol-binding protein 4 (RBP4) [26] is positively correlated to a higher concentration of IR. In addition, the delivery of fetuin-A, fetuin-B, and selenoprotein P (SeP) into the blood has autocrine, paracrine and endocrine activities, which promote the development of atherosclerosis by regulating endothelial dysfunction and infiltrating inflammatory cells of the vessel wall, causing multiple metabolic disorders [26, 27]. As a result of continuously increased secretion of hepatokines, the disorders associated with the generation and decomposition of glycogen along with the incidence of DM, CVD, and NAFLD rise [9, 26, 27].

### 2.4. Management of DM and NAFLD/ALD

The prevalence of NAFLD increases to approximately 70-75% among diabetic patients [14]. NAFLD and T2DM increase the incidence of liver cancer and CVD, while regulation of NAFLD decreases the risk of DM and other extrahepatic complications [9].

Either excess generation of peroxisome proliferator-activated receptor-*γ* (PPAR-*γ*) or underexpression of hepatocyte apoptosis participates in increasing the sensitivity of insulin [19], which could be achieved by regular improvement of NAFLD via reduced content of lipids in the liver and increased aliphatic acid *β*-oxidation [19, 25]. Physical exercise to lose weight and obtain good control on blood glucose could improve the levels of some antioxidant enzymes and mediators of anti-inflammation. These interventions further inhibit the overexpression of ROS and OS among NAFLD patients [19] and achieve maximum control of disease progression. Treatment for regulating intestinal flora decreases the susceptibility of NAFLD and chronic vascular complications of DM [11].

## 3. The Interrelation between DM and Hepatitis C Virus- (HCV-) Induced Liver Diseases

### 3.1. Epidemiological Characteristics of HCV-Related Diseases

HCV is a significant health issue affecting humans globally and has a genetically high diversity of seven genotypes and more than 80 subtypes. The global rate of genotypes 1-6 is 45%, 13%, 22%, 13%, 1%, and 2%, respectively, while the ratio of genotype 7 is unclear [28]. About 1.5 million people die each year due to viral hepatitis caused by HCV, and the quality of life of patients is limited [28]. There is a 1%-5% risk among the chronic hepatitis C (CHC) population to develop cirrhotic diseases, and 1%-5% of these patients could progress to HCC with a history of infection lasting over 20-30 years [29]. There is a strong link between CHC and DM. Mounting evidence indicated that chronic HCV infection can elicit a series of extrahepatic reactions, such as endocrine disturbances, abnormal autoimmunity, and turbulence of metabolic disorders, inducing hypobetalipoproteinemia, steatosis, IR, impaired glucose tolerance, thyroid disease, gonadal dysfunction, and even tumors like mixed cryoglobulinemia and B-cell NHLs [30]. Among these complications, DM and IR are the general manifestations related to fibrosis development and similarly HCC with HCV infection, which further affect the morbidity and mortality of patients [30–32].

### 3.2. Interaction between DM and HCV-Induced Diseases

#### 3.2.1. Effect of DM on HCV-Induced Diseases

DM accelerates the progression of liver cirrhosis with HCV infection and attenuates antiviral effect. The incidence of all complications of cirrhosis is higher in diabetic CHC patients, which included the esophageal varices bleeding, ascites, hepatic coma, HCC, spontaneous bacterial peritonitis (SBP), and renal failure based on severe liver disease-like hepatorenal syndrome (HRS) and cirrhosis-related mortality [33]. When DM, metabolic syndrome, and NAFLD with HCV infection coexist, it more easily tends to deteriorate to fibrosis, cirrhosis, and HCC [34]. A study has reported diabetes as an independent prediction factor for cirrhosis (adjusted HR 1.9, 95% CI: 1.05-3.43, *P* = 0.03) [35].

Among the HCV-related cirrhotic patients, compared to nondiabetic patients, diabetic cirrhosis patients had more severe hepatic encephalopathy (HE) (35% mild, 60% severe vs. 58% mild, and 20% severe) (*P* = 0.007) and also had relatively serious liver diseases [36]. A retrospective study conducted in the USA showed that among patients with compensatory cirrhosis and diabetes, HE and acute renal failure were the most common complications and had higher HRs for decompensated clinical manifestations (ascitic fluid: HR 1.67, 95% CI 1.45-1.91; variceal hemorrhage: HR 1.72, 95% CI 1.58-1.87; and acute kidney failure: HR 1.54, 95% CI 1.44-1.65) and developing SBP and liver cancer when compared to those with only cirrhosis [37] A prospective study recruited 250 HCV-induced cirrhotic compensation patients, and the results indicate that DM patients are associated with a higher total death rate or hepatic transplantation with a subhazard ratio (sHR) of 2.2 (95% CI: 1.04-4.6) and decompensated liver manifestations with sHR of 1.9 (95% CI: 1.05-3.3) (*P* = 0.03) after adjustments [38]. DM among cirrhotic patients with an Ishak fibrosis score of 6 (*n* = 303) demonstrated a significantly elevated risk for HCC with HR 3.28 (95% CI, 1.35–7.97; *P* = 0.0087) [39]. Stable glucose control (HbA1c < 7.0%) in diabetic CHC patients reduced the occurrence of HCC [40].

#### 3.2.2. Effect of HCV-Induced Diseases on DM

Chronic HCV infection elicits a series of extrahepatic manifestations including endocrine dyscrasia or dysbolism, which might lead to increased incidence or death rates, affecting the progression of diabetes [41].

HCV-induced cirrhosis acts as an independent risk for the growing occurrence of T2DM [42]. The risk of T2DM for HCV cirrhotic patients is higher than that of patients without cirrhosis. Cirrhotic subjects are 1.5 times more likely to develop T2DM than those without cirrhosis (aHR = 1.47) [42].

The growing frequency of T2DM and fasting blood glucose (FBG) is associated with the degree of hepatic cirrhotic lesions with HCV infection. HCV infection is the leading predictor of T2DM, along with the fibrosis-4 (FIB4) category, elder age, and greater BMI [32].

#### 3.2.3. Effect of Sustained Virologic Response (SVR) on DM

Eliminating HCV infection improves the prognosis of HCV-induced diseases, including the occurrence of liver cancer and decreased liver-related mortality [43]. The effectiveness of antiviral treatment may improve an individual's immune system [44].

At present, direct-acting antiviral (DAA) treatment improves FBG and glycated hemoglobin (HbA1C) in diabetic CHC patients and declines the rate of T2DM and cardiovascular risk among CHC patients [45]. Subsequent to covariate adjustment, SVR might be an independent factor for reducing the risk of DM in the future. A retrospective study conducted on treating DM patients with DAA regimen showed a decreased rate of DM occurrence in patients with SVR (231/3748; 6.2%, aHR = 0.79; 95% CI: 0.65-0.96) [42].

Compared to those untreated by interferon therapy or direct-acting antivirals (DAAs), SVR patients have a reduced risk of diabetic complications, including acute ischemic heart disease (sHR: 0.36, *P* < 0.001), terminal stage of CKD (sHR: 0.46, *P* < 0.001), cerebral infarction (sHR: 0.34, *P* < 0.001), and retinal diseases (sHR: 0.24) [46].

#### 3.2.4. Interaction between Antiviral Treatment and IR

Chronic HCV infection and HCV-induced metabolic diseases influence each other closely, as the coexisting metabolic disorders with HCV infection increases the risk of HCC, developing into advanced fibrosis [47].

Elimination of HCV can change IR and improve insulin sensitivity, hindering IR-induced symptoms and complications [31]. A prospective case-control study included 68 sustained fibrotic subjects (F3-F4) infected by HCV-genotype 1 treated with DAAs and 65 were untreated. Majority of SVR patients had improved IR, and some achieved normal IR levels. IR improvement and decrease in HOMA-IR were highly associated with HCV clearance (*P* < 0.001); however, the fibrotic subjects still had IR [31].

### 3.3. The Probable Mechanisms

Hyperglycemia and hyperinsulinemia generate matrix proteins and additional fibrosis-related precursors by hepatic stellate cells (HSCs), which subsequently accelerate the progression of cirrhotic disease [48]. In addition, there are a series of physiological or pathological activities in DM, such as the activation of HSCs, inflammatory changes, apoptotic process, formation of new blood vessels, and capillary changes in the hepatic sinus, thus affecting the fibrotic and cirrhotic progression [49]. HCV itself also takes part in the development of diabetes. HCV mainly invades the liver cells to generate substances for endocrine regulation [50, 51]. There might be a correlation between HCV proteins and insulin signaling cascade [51]. Proinflammatory cytokines caused by HCV dissemination, such as interleukin 6 (IL-6), also refer to IR [52].

The proteins secreted by adipose tissue such as leptin and adiponectin assist in the regulation of glycometabolism, lipometabolism, and energetic metabolism [30, 53]. Also, the particular adiponectin could improve insulin sensitivity. Upregulated secretion of tumor necrosis factor-*α* (TNF-*α*), and enhancive suppressor of cytokine signaling 3 (SOCS3) [53] lead to increased expression of ROS and other proinflammatory cytokines, affecting insulin signaling and cell dysfunction [54].

In addition, HCV also causes dyslipidemia. Reduced inhibition of insulin on hepatic glucose production (HGP) and accelerated tissue decomposition resulted in enhancive FFAs, further causing hyperglycemia and hypertriglyceridemia. This progression participates in the development of DM and also in notable systemic cardiovascular morbidity and mortality [55].

### 3.4. Management of DM in HCV-Related Diseases

At present, DAAs are the first choice for treating HCV. Better control of HCV-induced cirrhosis can effectively minimize the adverse consequences of DM, and their risk factors related to DM, improving the diabetic clinical outcomes. The powerful and safe DAAs assist in a viral clearance rate of over 95%, and based on these results, the clearance of HCV might be realized in the future [51]. With the elimination of HCV, not only HCV replication but also remission of liver injury, metabolic disorders, and immunologic derangement are controlled [43, 44]. Also, the liver cancer and liver-related mortality are decreased [43]. Indicators decline the rate of T2DM as well as cardiovascular risk, improving insulin sensitivity and *β*-function and decreasing FBG, C-peptide, HOMA-IR, and HbA1C [45, 51]. In the whole course of DAA treatment or during the 12-week follow-up period, glucose control in T2DM patients by oral medicine does not reduce the dosage of drugs, but there was a decrease in insulin use among those who underwent treatment by insulin [32].

## 4. The Interrelation between DM and Hepatitis B Virus- (HBV-) Related Diseases

### 4.1. Epidemiological Characteristics of HBV-Related Diseases

As mentioned above, cirrhosis is a very common cause of mortality worldwide. In contrast with Western and industrialized nations, the majority of the cirrhotic population that has HBV in Asian countries, especially in China, has neither ALD nor NAFLD [7]. Advanced fibrosis and the precursor of cirrhosis can occur before antiviral therapy in chronic hepatitis B (CHB) patients [56]. In 2015, about 900,000 deaths were HBV-infected diseases, including 450,000 cirrhosis and its complications, with a detailed population of 0.35 million of HCC and 0.1 million of acute HBV [7]. With the distribution of vaccines, the prevalence of HBV disease has gradually decreased [57]. Either chronic HBV or DM increases the risk of cirrhosis. A Chinese study suggested that diabetes is one of the hazardous factors of liver cancer among chronic HBV-infected patients, along with the risk factors such as cigarette smoking and high value of HBV [58]. HBV, diabetes, and IR interact with each other. IR plays a vital role in DM and metabolic syndrome. HBV infection could affect lipid metabolism. Crescent studies have shown that diabetes can affect the progression of liver diseases [59].

### 4.2. Interaction between DM and HBV-Related Diseases

#### 4.2.1. Effect of DM on HBV-Induced Diseases

DM increases the risk of cirrhotic hazard, especially in males [60]. The occurrence of diabetes, as well as obesity and metabolic syndrome in association with HBV infection, not only aggravates hepatic injury characterized by elevated alanine aminotransferase elevation but also makes a rapid development in hepatopathy, just like generation and progression of cirrhosis or even liver tumor [58, 61]. A prospective study conducted in China suggested that DM and hyperglycemia were connected with elevated risks of liver cancer and HBV-related liver diseases even in CHB patients with normal blood glucose metabolism. Along with elevated plasma glucose levels per 1 mmol/L, cirrhosis or HCC hazard in diabetic HBV-infected population would be elevated (adjusted hazards ratios 1.04 and 1.07, respectively) [61]. The effect of hypoglycemic therapy might influence the outcomes of HBV cirrhosis. A cohort study between 1997 and 2009 in Taiwan in chronic HBV patients showed that a poor response to insulin and/or sulphonylurea (HbA1C ≥ 7.0%) has prognosticated the occurrence of cancerous or cirrhotic complications (*P* < 0.05) [62].

To explore the effects of T2DM on HBV-related cirrhosis with morbidity and mortality, Hsiang et al. have revealed that the risk of liver cancer was increased (*P* = 0.006) among DM subjects with HBV-related cirrhosis. DM influences the clinical outcomes of HBV cirrhotic patients and acts as predictors of severe liver diseases such as HCC (HR 2.36, 95% CI 1.14-4.85, *P* = 0.02), decompensatory complications (HR 2.04, 95% CI 1.16-3.59, *P* = 0.01), and death rate or orthotopic liver transplantation (OLT) [62]. New-onset diabetes acts as an independent HBV-related cirrhotic prediction factor in multivariate analysis by COX regression (HR: 2.01; 95% CI: 1.39, 2.91) [60].

#### 4.2.2. Effect of HBV-Related Diseases on DM

The degree of inflammation and fibrosis is relevant to DM risk. The risk of T2DM for individuals developing HBV cirrhosis was higher than that for those without cirrhosis (odds ratio (OR) = 1.74; 95% CI 1.43-2.13) [63]. In the period from 1998 to 2003, a case-cohort study of HBV patients showed a positive correlation between diabetes and fibrotic degree for hepatitis B e antigen (HBeAg)-negative among Greek subjects [64]. Another French cohort study enrolled chronic HBV subjects between 2008 and 2013, and multiple factor analysis results indicated that diabetes was regarded as a risk factor of advanced hepatopathy (aHR 1.40, 95% CI: 1.32-1.48) [59].

The period of CHB infection history is associated with the incidence of DM. Shen et al. have found a positive relationship between cirrhosis and diabetes risk in CHB patients with a long history (i.e., >5 years), quadrupling the risk for diabetes. The change of lifted gamma-glutamyl transpeptidase (GGT) levels of above 4-fold upper limit of normal (ULN) rather than ALT affected the diabetic risk after adjusting for miscellaneous variable factors [65]. In addition, there were high levels of triglycerides, FBG, HbA1c, and insulin secretions, with reduced density lipoprotein (HDL) cholesterol among diabetic patients [65].

The factors such as CHB familial aggregation, cirrhotic occurrence, and HBeAg-positive status as well as higher HBV DNA (above 106 copies/mL) alleviated the OR of T2DM progression by logistic regression analysis, with OR values of 2.36 (95% CI 1.18-4.71; *P* < 0.05), 1.66 (95% CI 1.02-2.70; *P* < 0.05), 1.81 (95% CI 1.21-2.70; *P* < 0.01), and 2.84 (95% CI 1.73-4.66; *P* < 0.001) [65].

#### 4.2.3. Effect of Antiviral Treatment on DM

Along with DM, higher BMI, lower FGF-21 (*P* = 0.055), and higher AFABP, together with HOMA-IR levels (*P* < 0.001) showed correlation with more serious fibrosis/cirrhosis during antiviral therapy in people infected with CHB and received a 3.7-fold high risk of more serious fibrosis and cirrhosis [66]. Through the antiviral treatment of HBV, we may find a new way to regulate the glycometabolism and lipid metabolism [67].

Vaccination is the most basic measure to prevent HBV infection [57]. The incidence of HCC was obviously decreased among children aged 6-19 years who had vaccinations against HBV than for those who did not have vaccinations in the neonatal period. Also, nonstandard vaccination process was related to the occurrence of liver cancer [68].

Reduction of cirrhosis complications can be done by effectively suppressing virus proliferation via long-run application of nucleoside analogue therapy. Through propensity score matching models for age, gender, lipid-lowing treatment, presence of comorbid dyslipidemia and diabetes mellitus, compared to those patients treated on ETV, tenofovir disoproxil fumarate- (TDF-) treated patients benefitted from a 20% decrease of lipoprotein lipid profile components, including reduced application of lipid occurrence of concomitant lipid metabolism disorder of diabetes mellitus. TDF in patients benefitted from lipoprotein lipid profile components by decreasing the levels in different degrees, including the total cholesterol (TC) and low density lipoprotein cholesterol. These antiviral effects reduced the severity of lipid metabolism, improving the prognosis of atherosclerosis and CVDs associated with DM outcomes [69].

### 4.3. The Probable Mechanisms

The progression of inflammation and fibrosis induced by HBV leads to glycemia and might in turn participate in the progression of DM, as well as liver dysfunction, which in contrast promotes IR. In addition, HBV replication in different organs and tissues is involved in the development of diabetes. Distinct from HCV, HBV can replicate in other organs or tissues like the pancreas and bile. After replication in the pancreas, the *β*-cell function is damaged by HBV, further leading to disturbances in serum glucose metabolism [5]. Decreased hepatocyte masses or portosystemic shunting induces hyperinsulinemia and produces resistance to insulin via reduced expression of the insulin receptor. And further increases in the requirement of pancreatic insulin secretion eventually lead to diabetes [70].

Anabolism and catabolism are the two major categories of intrahepatic cellular glucose metabolism, which consist of the gluconeogenic pathway, aerobic glycolysis, and the pentose phosphate pathway [67]. The improved OS by hyperglycemia might be a concern associated with the seriousness of CHB-induced liver disease [67]. Any factor at the molecular level that affects the metabolic procession above is associated with the occurrence of DM.

As a regulator of gluconeogenesis, HBV X (HBx) [71] protein plays a vital role in promoting gluconeogenesis. Obviously, upregulation of the HBx gene for key enzymes participates in the gluconeogenic pathway, for instance, phosphoenolpyruvate carboxykinase (PEPCK) along with glucose-6-phosphatase (G6Pase) [67, 72]. Meanwhile, the increasing glucose generation is accompanied by elevated HBx expression, which is thought to be adjusted by means of the nitric oxide (NO)/JNK signaling pathway [67]. Pre-S2 protein inhibits insulin-receptor gene expression, inducing IR after elevating the secretion of soluble tumor necrosis factor receptors, which assist in controlling and adjusting gluconeogenic progression [5]. Several regulators of HBV expression are linked to lipid metabolism and glycometabolism, like hepatocyte nuclear receptors (HNFs) and others [73]. Bile acids are mainly synthesized from cholesterol in the liver, dominantly affecting the progression of digestion and absorption of lipids, along with the activation of farnesoid X receptor (FXR) [67, 73]. This process virtually forms a connecting link between HBV infection and metabolic syndrome. A few hepatic steatosis-related adipokines are associated with HBV fibrosis/cirrhosis, including the adipocyte fatty acid-binding protein (AFABP), which generates not only adipocytes and macrophages but also Kupffer cells [66].

### 4.4. Management of DM and HBV-Related Diseases

With the distribution of vaccines, the prevalence of HBV disease has gradually dropped [74]. In addition to antiviral treatment, HBV vaccination improved liver diseases and HCC [60], and the immunization has become a more successful one, easily reducing the incidence of diabetes [5]. Compared to healthy individuals, the rate of progression of DM among HBsAg carriers is nearly 3-fold (32.9%) when compared to those with HBsAg-negative [75]. As diabetics are more susceptible to HBV infection, adults aged 19 to 59 without vaccination should undergo vaccination for hepatitis B to avoid HBV infection and further HBV-related deterioration of DM [76].

Inhibition of virus replication and serological conversion of HBeAg through effective antiviral therapy reduce the onset of DM and reduce the control of serious complications associated with diabetes by improving the status of cirrhosis. Besides the suppression of viral replication, oral antiviral treatment benefits the clearance of necroinflammatory activity induced by chronic HBV infection, reduces vertical transmission from maternal to infant, halts or delays the progression of CHB-induced fibrosis/cirrhosis, and reduces the occurrence of HCC, further improving the patients' living conditions and quality of life [57].

## 5. Conclusion

Over the last few decades, the number of patients with diabetes around the world has increased. By 2030, the global rate of morbidity for adult diabetes patients could increase from 6.4% to 7.7% [77]. DM and its associated complications reduce the patients' quality of life, shorten the life expectancy, increase the mortality, and aggravate the economic burden [78]. The liver is regarded as the main organ for maintaining glucose metabolism [9, 70].

DM influences the clinical outcome of cirrhotic patients. Cirrhosis and diabetes interact and deteriorate each other's conditions. Diabetic patients have an increased risk of decompensated complications of cirrhosis and liver diseases, which influence their mortality rate [6, 62]. Meanwhile, hepatic dysfunction increases the occurrence of DM and diabetic complications, which conversely influences the prognosis of DM.

Management of DM includes application of oral hypoglycemic agents and insulin and improvement of lifestyle therapy, such as appropriate physical exercise, ample sleep, and quitting smoking. These changes could improve DM and reduce the risk of DM complications [2]. Mounting evidences have suggested that controlling liver cirrhosis, regardless of the pathogenesis, through antiviral treatments for virtual liver diseases lowers the severity of complications associated with diabetes. In view of the relationship between DM and liver diseases, proper management of diabetes and DM-related complications is of utmost importance.
